# Type I Interferon Signature in Primary Antiphospholipid Syndrome: Clinical and Laboratory Associations

**DOI:** 10.3389/fimmu.2019.00487

**Published:** 2019-03-15

**Authors:** Eleni Palli, Evrydiki Kravvariti, Maria G. Tektonidou

**Affiliations:** Joint Academic Rheumatology Program, First Department of Propaedeutic Internal Medicine, School of Medicine, National and Kapodistrian University of Athens, Athens, Greece

**Keywords:** antiphospholipid syndrome, systemic lupus erythematosus, type I Interferon signature, type I Interferon score, antiphospholipid antibodies, anti-b2-glycoprotein I antibodies, hydroxycloroquine

## Abstract

**Background:** Increased expression of type I interferon (IFN)-regulated genes has been described in blood and tissue cells from patients with systemic lupus erythematosus (SLE) and other rheumatic disorders. Only isolated studies have examined the type I IFN gene expression in antiphosholipid syndrome (APS), while efforts to evaluate associations with APS-related factors are scarce.

**Objective:** Our aim was to investigate the type I IFN signature in patients with primary APS (PAPS), SLE/APS, and SLE in comparison with healthy controls, and to evaluate associations with disease-related characteristics.

**Methods:** We measured the type I IFN score, derived from relative expressions of three IFN-inducible genes (MX-1, IFIT-1, and IFI-44) in peripheral blood mononuclear cells from 55 patients with PAPS, 34 with SLE/APS, 48 with SLE, and 28 controls. In patients with PAPS, we performed multivariate regression to examine associations of type I IFN score with their clinical, laboratory and treatment characteristics.

**Results:** Type I IFN score was increased in all patient groups vs. controls (*p* = 0.028, *p* = 0.027, *p* = 0.028 for PAPS, SLE/APS, and SLE, respectively). IFI-44 had the most pronounced expression. In patients with PAPS, multivariate linear regression revealed positive associations of type I IFN score with female gender (b-coefficient = 0.49; 95% CI 0.04, 0.94; *p* = 0.034) and IgG or IgM anti-β2GPI antibodies (b-coefficient = 0.53; 95% CI 0.10, 0.96; *p* = 0.017), and negative associations with age (b-coefficient = −0.02/year; 95% CI −0.04, −0.01; *p* = 0.027) and hydroxychloroquine use (b-coefficient = −0.51; 95% CI-0.96, −0.06; *p* = 0.027).

**Conclusion:** Type I IFN score is increased in PAPS and correlated positively with anti-β2GPI antibodies and negatively with hydroxychloroquine use.

## Introduction

Antiphospholipid syndrome (APS) is a systemic autoimmune disease characterized by recurrent venous and/or arterial thrombotic events and/or obstetric complications and the presence of antiphospholipid antibodies (aPL) including anti-cardiolipin antibodies (aCL), anti-beta2-glycoprotein I antibodies (anti-β2GPI), and lupus anticoagulant (LA) (1). APS occurs either as primary APS (PAPS) or secondary APS, when it is associated with other autoimmune diseases, mainly systemic lupus erythematosus (SLE/APS). Approximately 30–40% of SLE patients have positive aPL and almost one third of them develop APS (1, 2). In addition to well-characterized thrombotic events, other manifestations such as thrombocytopenia, livedo reticularis, valvular disease, APS nephropathy, or neurological disorders such as epilepsy and cognitive dysfunction may occur in APS, defined as non-criteria APS manifestations (3–5). Accordingly, in addition to thrombogenic mechanisms, aPL-mediated inflammatory processes have also been identified in APS, and emerging therapies associated with these inflammatory pathways include hydroxychloroquine, B-cell targeted therapy, complement inhibition, peptide therapy, mTOR inhibitors, and others (6).

Type I interferons (IFNs) are cytokines that have various effects on innate and adaptive immune cells and have been implicated in the pathogenesis of a number of systemic autoimmune diseases including SLE, Sjögren's syndrome, rheumatoid arthritis, systemic sclerosis and myositis (7–11). Gene expression profiling data from patients with SLE have shown high messenger RNA transcripts of genes regulated by type I IFN, also known as IFN signature, that correlated with clinical and laboratory indices of lupus activity in several studies (12, 13). These findings have spurred research evaluating type I IFN-blocking agents as therapeutic alternatives in SLE (14). Only a few studies have addressed the question of IFN-inducible gene expression in PAPS and SLE/APS (15–17). However, most studies had a small sample size and only one attempted to correlate the type I IFN signature to disease-related characteristics of patients with PAPS (17).

The aim of this study was to compare the type I IFN signature in peripheral blood mononuclear cells (PBMCs) from patients with PAPS, patients with SLE/APS, patients with SLE not fulfilling the classification criteria for APS, and healthy individuals. We also evaluated potential associations between type I IFN signature and several clinical, laboratory and treatment characteristics of patients with PAPS.

## Materials and Methods

Consecutive adult patients with PAPS and age-matched patients with SLE/APS and SLE/non-APS followed in our department, were included in the study. Healthy individuals matched to PAPS patients by age distribution were recruited using brochures in the hospital and local community centers. Patients with APS (either PAPS or SLE/APS) fulfilled the updated Sapporo classification criteria (1), and patients with SLE met the updated ACR classification criteria for SLE (18). Candidates with active infection or hospitalization within the previous month, pregnancy, or history of malignancy, were excluded. The study protocol was approved by the local IRB (“Laikon Hospital Scientific Council”) and all participants provided written informed consent.

At the study visit, all patients underwent thorough clinical and laboratory evaluation and their medical records were reviewed. For patients with APS, we recorded the details of prior thrombotic events (number, type, location) and/or obstetric complications establishing the diagnosis of APS, as well as the presence of non-criteria clinical manifestations as defined by the updated Sapporo classification criteria for APS (1). For patients with SLE, we recorded disease activity using the Systemic Lupus Erythematosus (SLE) Disease Activity Index-−2000 (SLEDAI-2K), as well as history of major SLE complications, including nephritis and CNS involvement. Medication use was reviewed and recorded including corticosteroids, hydroxychloroquine, immunosuppressives (cyclophosphamide, azathioprine, mycophenolate mofetil, methotrexate, leflunomide, and cyclosporine), antiplatelets, oral anticoagulants, and statins. Patients receiving biologic agents such as B cell depletion and adalimumab were excluded.

Blood samples were drawn from patients for immunologic tests including antinuclear antibodies, anti-dsDNA, anti-Sm, anti-Ro/SSA and anti-La/SSB antibodies, C3 and C4 levels, aCL and anti-β2GPI antibodies of IgG and IgM isotype using standard ELISA (1), and LA measured according to Scientific Standardization Subcommittee (SSC) on Lupus Anticoagulant/Phospholipid Antibodies guidelines (19). Additional 8–10 mL samples were collected from all participants for the isolation of PBMCs using Lymphoprep (Stem Cell Technologies) as the density gradient medium, according to the manufacturer's instructions.

Total RNA was extracted from PBMCs using Trizol reagent (Ambion, Life Sciences, USA) according to standard procedure. RNA concentration and quality was determined by spectrophotometry (Biospec Nano, Japan). Total RNA was transcribed into cDNA, which was then quantified by Quantitative Real-Time PCR (qRT-PCR) reaction as previously described (20). Three genes typically induced by type I IFN (10, 16, 20) were selected: myxovirus (influenza virus) resistance 1 (MX-1), interferon-induced protein with tetratricopeptide repeats 1 (IFIT-1) and interferon-induced protein 44 (IFI44). The levels of mRNA expression of type I IFN inducible-genes were determined based on the expression value of the glyceraldehyde phosphate dehydrogenase (GAPDH) reporter-housekeeping gene. Type I IFN score, was defined as the sum of relative expressions of MX-1, IFIT-1, and IFI-44 and calculated as previously described (20, 21). Type I IFN score was considered high if it exceeded the mean + 2^*^standard deviation (SD) value of the control group.

### Statistical Analysis

Between-group comparisons were tested using the Kruskal-Wallis and Mann-Whitney tests for continuous data, and Chi2 tests for nominal data. The Shidak-Holm method was used for correction of *p*-values for multiple comparisons. For patients with PAPS, we used the Mann-Whitney test to assess univariate associations of type I IFN score with nominal disease-related factors, and age as a binary variable, dichotomized on the median of PAPS group. We then applied linear regression to investigate multivariate associations of log-transformed type-I IFN score with clinical, laboratory and treatment characteristics of patients with PAPS. We applied the stepwise backward algorithm (*p* = 0.1) to construct the final multivariate linear regression model, starting from an initial model containing the PAPS-related parameters that demonstrated univariate associations with the type I IFN score at the 0.25 level and adjusting for age and gender. Statistical analysis was performed using STATA version 12.0 (College Station™, TX, USA) and Graph Pad PRISM software (La Jolla™, CA, USA).

## Results

We included in the analysis 55 patients with PAPS, 34 age-matched patients with SLE/APS and 48 with SLE/non-APS, and 28 healthy controls. Patient characteristics are shown in Table 1. Female predominance was more pronounced in the SLE group, followed by patients with SLE/APS. The group of PAPS patients characterized by more arterial thrombotic events, recurrent venous or arterial thromboses, and obstetric APS events, but fewer non-criteria APS manifestations than the SLE/APS group. Among patients with PAPS, 35% had positive ANA in low titers, but none had SLE-specific antibody positivity such as anti-dsDNA or anti-Sm antibodies. None had anti-Ro/SSA or anti-La/SSB antibodies.

**Table 1:** Baseline characteristics of study participant groups.

	**PAPS**	**SLE/APS**	**SLE**
	***n* = 55**	***n* = 34**	***n* = 48**
Age (years, mean ± SD)	44.6 ± 13.2	45.4 ± 11.5	47.4 ± 16.7
Female gender [*n*, (%)]	34 (62)	28 (82)	45 (94)
Disease duration (years, mean ± SD)	7.6 ± 7.5	11.3 ± 9.3	9.6 ± 7.7
Thrombotic events [*n*, (%)]	48 (87)	31 (91)	2 (4)
Venous thrombosis [*n*, (%)]	25 (46)	19(56)	1 (2)
Arterial thrombosis [*n*, (%)]	33 (60)	15 (44)	1 (2)
Both arterial and venous events [*n*, (%)]	10 (18)	4(12)	0 (0)
CVA [*n*, (%)]	18 (33)	12 (35)	0 (0)
Recurrent thrombotic events [*n*, (%)]	23 (42)	13 (38)	1(2)
Obstetric APS complications [*n*, (% among women)]	14 (41)	9(32)	0 (0)
Non-criteria APS manifestations [*n*, (%)]	18(33)	16 (47)	-
Corticosteroid use [*n*, (%)]	0 (0)	15 (44)	29 (60)
Immunosuppresives use [*n*, (%)]	0 (0)	18 (53)	19 (40)
Hydroxychloroquine use [*n*, (%)]	29 (53)	25 (74)	39 (81)
Antiplatelet use [*n*, (%)]	28 (51)	15 (44)	19 (40)
Statin use [*n*, (%)]	9 (16)	10 (29)	7 (15)
Oral anticoagulation [*n*, (%)]	48 (87)	32 (94)	1 (2)
LA positivity [*n*, (%)]	42 (76)	24 (71)	7 (15)
Anticardiolipin IgG positivity [*n*, (%)]	26 (47)	19 (56)	10 (21)
Anticardiolipin IgM positivity[*n*, (%)]	26 (47)	17 (50)	11 (23)
Anti-β2GPI IgG positivity [*n*, (%)]	22 (40)	11 (32)	6 (13)
Anti-β2GPI IgM positivity [*n*, (%)]	13 (24)	10 (29)	4 (8)
Double aPL positivity [*n*, (%)]	35 (64)	23 (68)	9 (19)
Triple aPL positivity [*n*, (%)]	22 (40)	14 (41)	5 (10)
Complement C3 levels (mean ± SD)	105 ± 25	92 ± 19	87 ± 26
Complement C4 levels (mean ± SD)	20 ± 8	16 ± 8	16 ± 8
Low complement levels [*n*, (%)]	7 (13)	8 (24)	13 (27)
Antinuclear antibodies [*n*, (%)]	19 (35)	29 (85)	39 (81)
SLEDAI (mean ± SD)	-	2.61 ± 1.92	3 ± 4.48
History of nephritis [*n*, (%)]	-	6 (18)	10 (21)
History of CNS involvement [*n*, (%)]	-	8 (24)	3 (6)

*SD, Standard Deviation; HC, Healthy Controls; APS, Antiphospholipid Syndrome; PAPS, Primary APS; SLE, Systemic Lupus Erythematosus; SLE/APS, Systemic Lupus Erythematosus-associated APS; CVA, Cerebrovascular accident; SLEDAI, SLE Disease Activity Index; CNS, Central Nervous System*.

**Figure 1:**
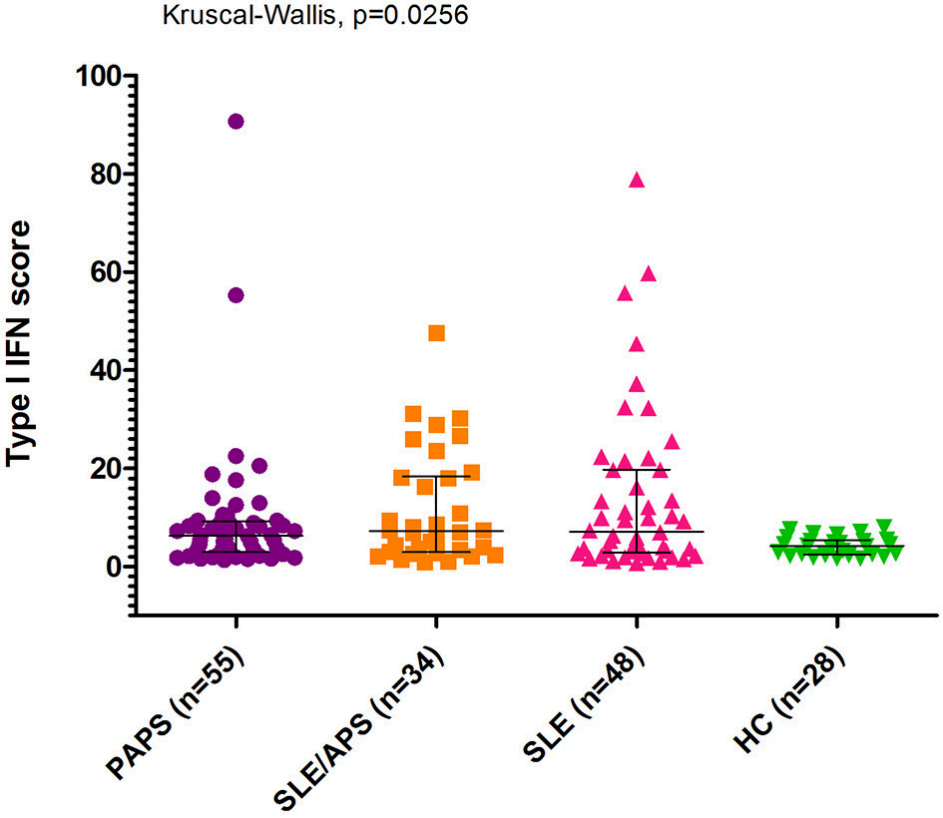
Type I interferon score measurements with median and interquartile range in patients with PAPS, SLE/APS, and SLE vs. healthy controls. IFN, Interferon; HC, Healthy Controls; APS, Antiphospholipid Syndrome; PAPS, Primary APS; SLE, Systemic Lupus Erythematosus; SLE/APS, Systemic Lupus Erythematosus-associated APS.

**Figure 2:**
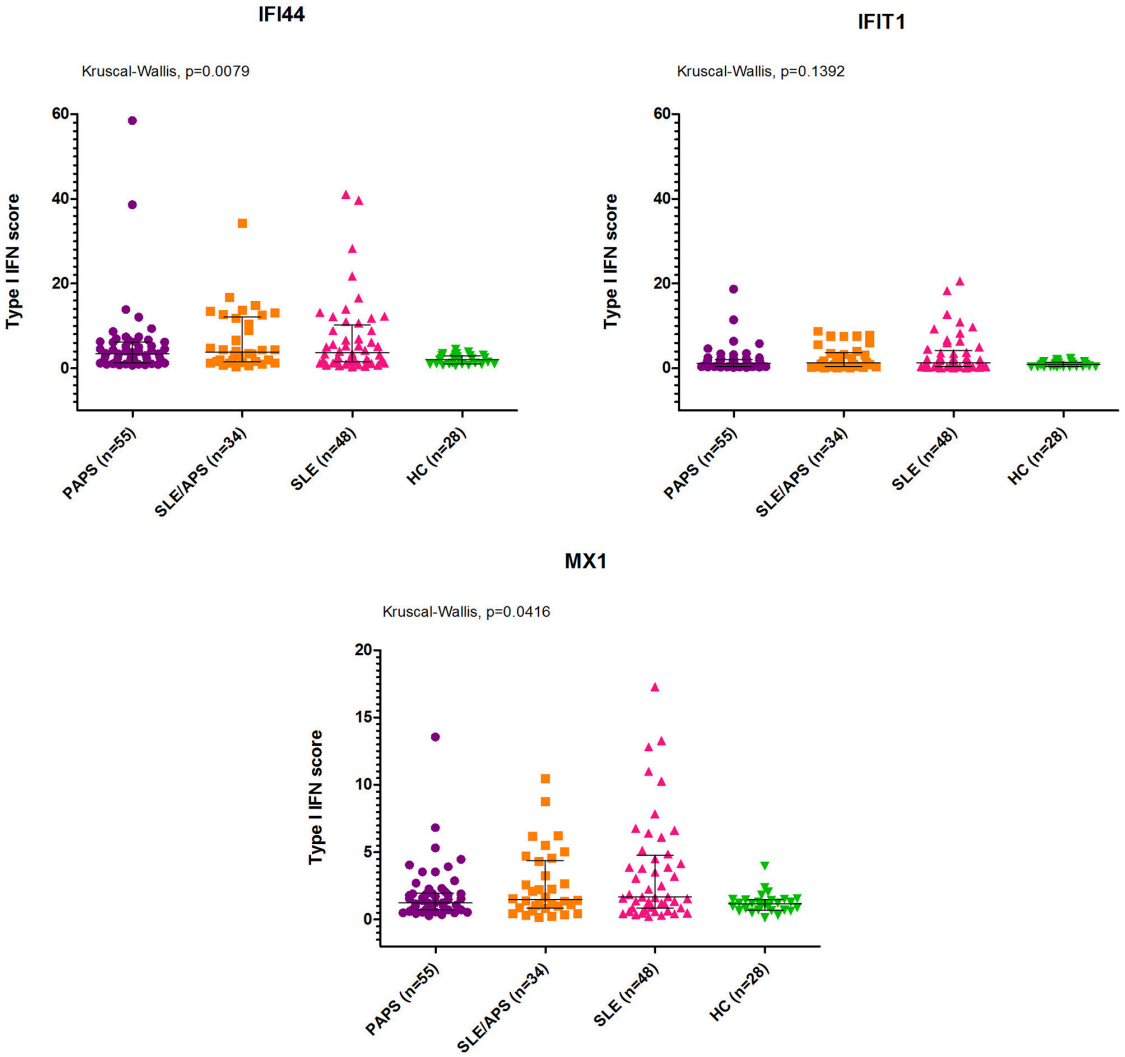
IFI44, IFIT1, and MX1 mRNA expression levels with median and interquartile range in PAPS, SLE/APS, and SLE patients vs. healthy controls. HC, Healthy Controls; APS, Antiphospholipid Syndrome; PAPS, Primary APS; SLE, Systemic Lupus Erythematosus; SLE/APS, Systemic Lupus Erythematosus-associated APS.

**Figure 3:**
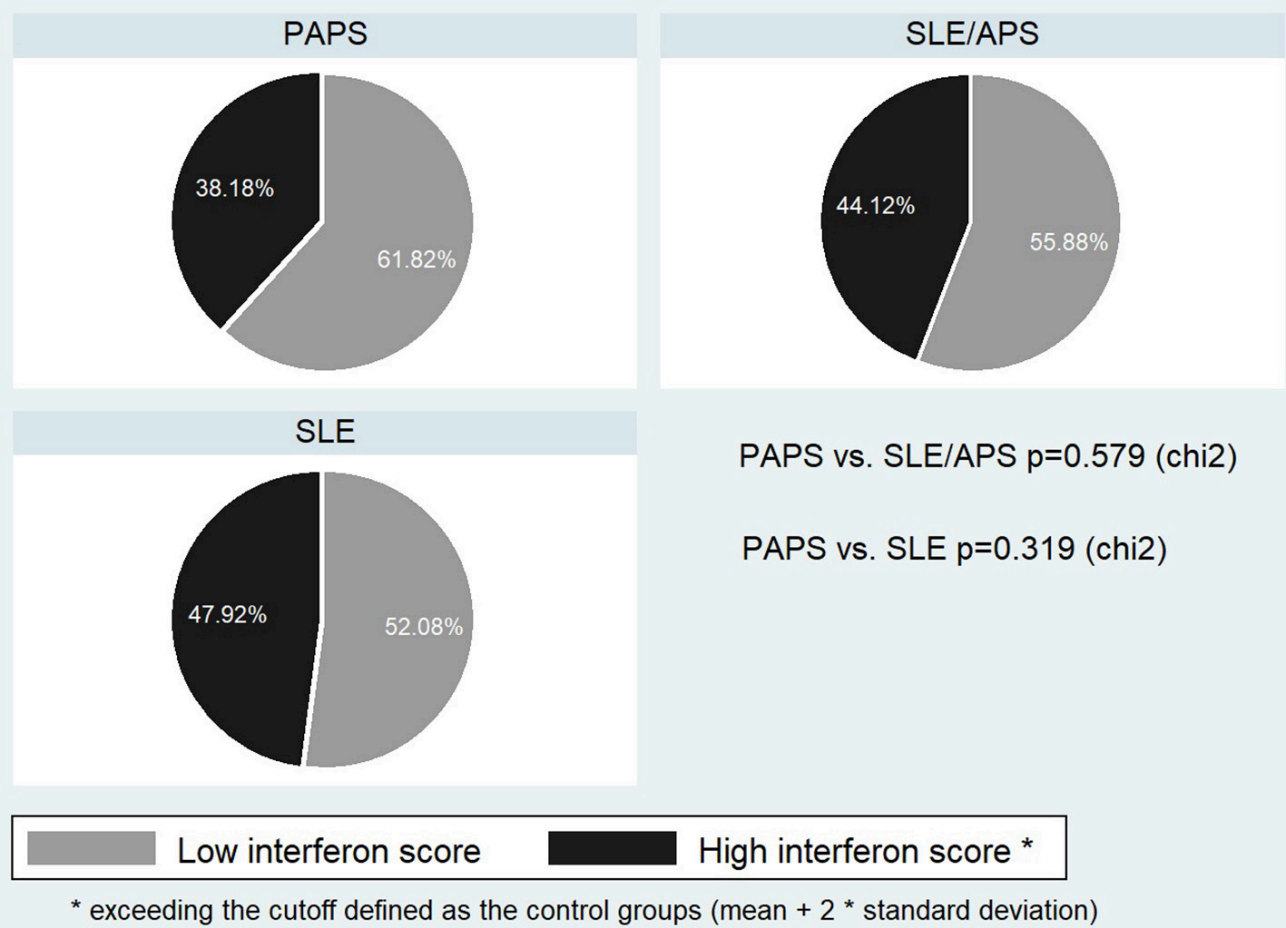
Frequency of high type I IFN score in PAPS, SLE, and SLE/APS groups. APS, Antiphospholipid Syndrome; PAPS, Primary APS; SLE, Systemic Lupus Erythematosus; SLE/APS, Systemic Lupus Erythematosus-associated APS.

The median type I IFN score was highest in patients with SLE/APS (7.2, IQR: 3.0–18.1), followed by patients with SLE (7.1, IQR: 2.7–19.7), PAPS 6.3, IQR: 3.0–9.2), and controls (4.2, IQR: 2.5–5.4) (Figure 1). Mann-Whitney tests for comparison of the type I IFN score in each patient group vs. controls yielded unadjusted *p*-values of 0.014 for PAPS, 0.009 for SLE/APS, and 0.018 for SLE, and multiple-comparison adjusted *p-*values of 0.028, 0.027, and 0.028, respectively. Among the three genes comprising the type I IFN score, differences between patients and controls were most pronounced in the fold expression of IFI-44 (Figure 2). A high type I IFN signature was observed in 47.92% of patients with SLE, 44.12% of SLE/APS, and 38.18% of patients with PAPS (Figure 3).

Among patients with PAPS, the type I IFN score in univariate analysis was associated with anti-β2GPI antibodies of either IgG or IgM isotype, with a trend for an association with triple aPL positivity (Table 2). In multivariate regression, estimates derived from linear regression constructed by applying a stepwise backward algorithm (*p* for removal = 0.1) to an initial model including age, gender, arterial events, aCL, antiβ2GPI, triple aPL, current use of hydroxycloroquine, and statins. The results of multivariate analysis (Table 3) showed that type I IFN score was significantly higher in females (b-coefficient = 0.49, 95% CI (0.04, 0.94), *p* = 0.034) and patients with medium-to-high IgG or IgM anti-β2GPI antibodies (b-coefficient = 0.53, 95% CI (0.10–0.96), *p* = 0.017), and lower with increasing age (b-coefficient = −0.02 per year, 95% CI: (−0.04, −0.01), *p* = 0.027) and hydroxychloroquine use (b-coefficient = −0.51, 95% CI (−0.96, −0.06), *p* = 0.027).

**Table 2:** Univariate associations between type I interferon score and disease-related factors in patients with PAPS.

**Disease-related factor**	**Type I interferon score median, (interquartile range)**		*p*-value^*^
	**Factor absent**	**Factor present**	
Age >44 years#	6.45 (3.63–9.38)	5.13 (2.11–8.41)	0.125
Female gender	4.99 (2.54–8.41)	6.82 (3.56–9.38)	0.239
Arterial thrombotic events	7.55 (2.98–12.97)	5.30 (2.95–8.41)	0.223
Non-criteria APS manifestations	6.45 (2.54–9.36)	5.28 (3.56–8.38)	0.733
Recurrent thrombotic events	6.44 (2.66–9.88)	5.30 (3.56–8.41)	0.682
Obstetric APS complications	6.27 (3.56–17.61)	4.99 (2.37–8.41)	0.277
aCL positivity	3.05 (1.82–9.18)	6.45 (3.63–8.92)	0.122
Anti-β2GPI positivity	4.99 (1.94–8.7)	8.00 (4.30–9.37)	0.045
LA positivity	7.21 (2.17–8.70)	5.92 (3.55–9.38)	0.663
Triple aPL positivity	4.99 (2.11–8.78)	7.54 (5.03–9.38)	0.057
Hydroxychloroquine use	8.00 (4.15–9.38)	5.30 (2.95–8.38)	0.231
Antiplatelet use	6.44 (2.54–8.78)	5.92 (3.55–9.88)	0.602
Statin use	6.83 (3.55–9.36)	3.63 (1.94–5.30)	0.101

* *P-value derived from Mann-Whitney U*.

#Cut off selected to coincide with the median age in the PAPS group.

Anti-β2GPI, anti-beta2-glycoprotein I antibody; aCL, anti-cardiolipin antibody; aPL, antiphospholipid antibody; APS, antiphospholipid syndrome; PAPS, primary APS; SLE/APS, Systemic Lupus Erythematosus-associated APS.

**Table 3:** Multivariate linear regression model of clinical and laboratory determinants of log-transformed type I interferon score in patients with PAPS.

**Parameter**	**β coefficient**	**95% confidence interval**	***P*-value**
Age (per year)	−0.02	−0.04, −0.02	0.027
Female gender	0.49	0.04, 0.94	0.034
Hydroxychloroquine use	−0.51	−0.95, −0.06	0.027
Anti-β2GPI positivity	0.53	0.10, 0.96	0.017

*Anti-β2GPI, anti-beta2-glycoprotein I antibody; PAPS, primary APS*.

## Discussion

In this study, we found that patients with PAPS had high type I IFN score relative to controls, and similar to that in SLE and SLE/APS patients. Patients with PAPS with medium-to-high anti-β2GPI antibody titers of either IgG or IgM isotype had higher IFN scores, whereas those treated with hydroxychloroquine had lower scores, after adjusting for age and gender and other APS-related factors.

The findings of high type I IFN score in patients with PAPS vs. controls, and in comparable levels to that in SLE and SLE/APS patients, indicate that inflammatory pathways involving type I IFN may be implicated in the pathophysiology of APS, independently of SLE co-existence. Our findings are congruent to those of previous studies on type I IFN signature in PAPS. Bernales and colleagues reported for the first time high type I IFN signature in a small series of 13 PAPS and 17 SLE patients compared to controls (15). Grenn and colleagues found elevated levels of the IFN-inducible genes IFIT-1, IFI44, and PRKR in PBMCs and sera from 42 PAPS patients, and in serum samples from an independent cohort of 26 patients with PAPS (16). Van den Hoogen and colleagues showed that type I IFN score was higher in 24 patients with PAPS than controls, but lower compared to 47 patients with SLE and 28 with SLE/APS (17). Recently, Knight and colleagues demonstrated a proinflammatory gene expression signature in PAPS using RNA sequencing, mainly driven by up-regulation of type I IFN-inducible genes and more specifically by high transcription of IFIT-1 and MX-1 (22), which was also highly expressed in our patients with PAPS.

In our PAPS patients, anti-β2GPI antibodies were significantly correlated with type IFN scores. Our findings are in accordance with those of Grenn and colleagues, showing a significant correlation between anti-β2GPI positivity and elevated levels of IFIT-1 and IFI44 in PBMCs of PAPS patients (16). Basic research findings also support that aPL can induce IFNα production in cell cultures from pDCs from patients with APS (23). Furthermore, antibodies to β2GPI, especially to domain I of β2GPI, emerge in current research as one of the main pathogenic autoantibody subsets in APS associated with several phenotypes in the APS spectrum (24).

We also found that hydroxychloroquine use is associated with significantly lower type I IFN scores in patients with PAPS, confirming the results by van den Hoogen and colleagues (17). Hydroxychloroquine can attenuate the IFN signature via modulating Toll-like receptor (TLR) signaling and blocking neutrophil extracellular traps activity (22, 25). Hydroxychloroquine in patients with PAPS is the object of rigorous investigation, and promising evidence currently exists for its role in aPL production (26), and prevention of thrombotic (6, 27, 28) and obstetric event recurrences (29–31).

Although anticoagulation is the mainstay of treatment for APS, several patients experience recurrent thrombotic events and pregnancy morbidity despite the use of conventional treatments. In addition, anticoagulation is largely ineffective for non-criteria APS-related manifestations (5), which may be better addressed by alternative treatments targeting inflammatory pathways. Grenn and colleagues recently demonstrated that the impaired ability of endothelial progenitors to differentiate into endothelial cells was reversed by a type I IFN receptor-neutralizing antibody (16). Further research into IFN-inducible gene expression (including both type I and II IFN signature) and inflammatory dysregulation in APS could pave the way for new emerging therapies in APS potentially including interferon-blocking agents.

Our study has certain limitations. Due to the rarity of APS, the sample size was not adequate to support multivariate models which would include all individual APS-related parameters potentially affecting the type I IFN score, and power to detect weaker associations may be limited. Nevertheless, our results are derived from a cohort of well-characterized and closely monitored patients, and this is the first study to show a multivariate association between high IFN signature and anti-β2GPI antibody positivity in patients with APS, while adjusting for age, gender, and other significant disease-related factors.

In conclusion, patients with PAPS are characterized by a high type I interferon signature, which seems to be more pronounced in those with anti-β2GPI positivity, and limited in those receiving hydroxychloroquine treatment. Our findings highlight the need for further investigation of IFN pathways in PAPS, aiming to elucidate their role in the pathogenesis of thrombotic and non-thrombotic complications and investigate their potential as therapeutic targets for selected patients.

## Data Availability

The datasets generated for this study are available on request to the corresponding author.

## Ethics Statement

This study was carried out in accordance with the recommendations of local IRB (“Laikon Hospital Scientific Council”) with written informed consent from all subjects. All subjects gave written informed consent in accordance with the Declaration of Helsinki. The protocol was approved by the Laikon Hospital Scientific Council.

## Author Contributions

MT conceived the original idea, supervised and interpreted the findings of this work, and the manuscript writing. EP performed the experiments and wrote the manuscript. EK contributed to sample preparation, performed the statistical analysis, and helped to draft the manuscript. All authors provided critical feedback and helped shape the research, analysis, and manuscript.

### Conflict of Interest Statement

The authors declare that the research was conducted in the absence of any commercial or financial relationships that could be construed as a potential conflict of interest.
